# Care of the Teeth

**Published:** 1916-11

**Authors:** 


### Care of the Teeth.

A recent investigation made by the United States Public Health Service in connection with studies of rural school children showed that 49.3 per cent had defective teeth, 21.1 per cent had two or more missing teeth, and only 16.9 per cent had had dental attention. Over 14 per cent never used a toothbrush, 58.2 per cent used one occasionally, and only 27.4 per cent used one daily.

Defective teeth reduce physical efficiency. Dirty, suppurating, snaggle-toothed mouths are responsible for many cases of heart disease, rheumatism, and other chronic affections.

The children are not responsible for the neglected state of their teeth. The ignorant and careless parent is to blame for this condition—a condition which hampers mental and physical growth and puts a permanent handicap on our future citizens. School teachers can and are doing much in inculcating habits of personal cleanliness on the rural school child but this will fail of the highest accomplishment unless parents co-operate heartily and continuously.

The family physician can render no more helpful service to parents than to caution them occasionally about the proper care and preservation of the teeth of the children. Especially as the physician can have no pecuniary interest in such advice, he should feel no delicacy in giving it, and in most cases it will be most gratefully received and adopted.

